# *Saccharomyces paradoxus* Transcriptional Alterations in Cells of Distinct Phenotype and Viral dsRNA Content

**DOI:** 10.3390/microorganisms8121902

**Published:** 2020-11-30

**Authors:** Bazilė Ravoitytė, Juliana Lukša, Vyacheslav Yurchenko, Saulius Serva, Elena Servienė

**Affiliations:** 1 Laboratory of Genetics, Institute of Botany, Nature Research Centre, Akademijos str. 2, 08412 Vilnius, Lithuania; juliana.luksa@gamtc.lt; 2 Life Science Research Centre, Faculty of Science, University of Ostrava, Chittussiho 10, 710 00 Ostrava, Czech Republic; vyacheslav.yurchenko@osu.cz; 3 Martsinovsky Institute of Medical Parasitology, Tropical and Vector Borne Diseases, Sechenov University, Malaya Pirogovskaya str. 20, 119435 Moscow, Russia; 4 Department of Biochemistry and Molecular Biology, Institute of Biosciences, Vilnius University, Saulėtekio al. 7, 10257 Vilnius, Lithuania; saulius.serva@gf.vu.lt; 5 Department of Chemistry and Bioengineering, Vilnius Gediminas Technical University, Saulėtekio al. 11, 10223 Vilnius, Lithuania

**Keywords:** *Saccharomyces paradoxus*, dsRNA viruses, host gene expression, RNA-Seq

## Abstract

Killer yeasts are attractive antifungal agents with great potential applications in the food industry. Natural *Saccharomyces paradoxus* isolates provide new dsRNA-based killer systems available for investigation. The presence of viral dsRNA may alter transcriptional profile of *S. paradoxus*. To test this possibility, a high-throughput RNA sequencing was employed to compare the transcriptomes of *S. paradoxus* AML 15-66 K66 killer strains after curing them of either M-66 alone or both M-66 and L-A-66 dsRNA viruses. The *S. paradoxus* cells cured of viral dsRNA(s) showed respiration deficient or altered sporulation patterns. We have identified numerous changes in the transcription profile of genes including those linked to ribosomes and amino acid biosynthesis, as well as mitochondrial function. Our work advance studies of transcriptional adaptations of *Saccharomyces* spp. induced by changes in phenotype and set of dsRNA viruses, reported for the first time.

## 1. Introduction

*Saccharomyces cerevisiae* is one of the most extensively studied yeast species, being recognized as the main driver in various industrial fermentations, such as wine, beer, and bread production [1]. Its closest known relative, *Saccharomyces paradoxus*, is widespread in natural habitats and is commonly isolated from fermentative environments, often contributing to the aroma of wines [2,3,4]. The attractiveness of *Saccharomyces* spp. yeasts for the food industry is further increasing because of the killer toxin production. Killer yeast phenotype provide competitive advantages to the host and, thus, promotes their biocontrol application [5,6].

Yeast killer trait is often determined by persistent dsRNA viruses, providing a system for killer toxin production and maintenance. The L-A dsRNA virus of the family *Totiviridae* encodes proteins for viral capsid formation and viral genome replication, whereas M dsRNA employs these proteins for its own maintenance and codes for killer toxin with self-immunity feature [7]. As such, the L-A and M dsRNA viruses act in synergy with each other and the host cell. *Saccharomyces paradoxus* and *S. cerevisiae* are convenient models for the investigation of the relationship between dsRNA viruses and the host cell [8]. The importance of the host background in natural strains was revealed by different killer phenotypes of distinct strains bearing dsRNA viruses of the same type, explained by the differences in virus and host coevolution in different populations [9,10]. Incompatibility between *S. paradoxus* and *S. cerevisiae* mitochondria is considered as one of the main barriers to transmission of dsRNA viruses between species [11]. The relationship between dsRNA totiviruses and their *S. cerevisiae* hosts were uncovered by gene expression studies, supporting the idea that long-lasting coadaptation led to the moderate transcriptional responses following the elimination of the viral genome(s) [12,13]. Specific *S. cerevisiae* host cell lipidomic and transcriptomic adaptations were shown to depend on the quantity of the produced K1 killer toxin [14]. In addition to known *S. cerevisiae* dsRNA viruses, sequences of various *S. paradoxus* dsRNA viruses have been published recently [11,15].

Killing phenotype of industrial yeasts is a desirable trait and its stabile maintenance is important for the inhibition of food and beverage spoilage microorganisms, and preservation of the quality of the product. Curing of dsRNA has been employed as a tool to examine the stability and relationships of dsRNA viruses and the host cells. Application of various methods to cure *S. cerevisiae* yeasts of dsRNA viruses often induced the formation of respiration-deficient mutants that showed *petite* phenotype. The emergence of *petites* does not correlate with the loss of the killer phenotype [16,17,18]. Similarly, the inheritance of mitochondria and dsRNA viruses appear to be independent [19], yet their functions are interconnected (at least in *S. cerevisiae*) [12,20]. Disruption of *MAK* genes results in a loss of killer phenotype, however, elimination of mitochondrial DNA suppresses some of *mak* nuclear mutations [21]. In certain backgrounds, the *petite* cells demonstrated a stronger killer phenotype than their wild type counterparts [16]. We have previously reported that removal of dsRNA(s) from *S. cerevisiae* cells affects the transcription of genes related to mitochondrial functions, including respiration and ATP synthesis [12]. However, to the best of our knowledge, there are no published data linking mitochondria function and dsRNA viruses’ presence in *S. paradoxus*.

Mitochondrial functions are essential for the proper establishment of various cellular functions, including aerobic respiration, sporulation, and meiosis; thus, these processes are tightly interconnected in yeasts [22,23]. Similarly to *S. cerevisiae*, *S. paradoxus* cells proliferate mostly asexually [24]. Sexual reproduction in these species was also documented, however, diploid cells induce sporulation under certain conditions upon nutrient deprivation [25]. Sporulation is a complex process needed for survival and adaptation to changing environmental conditions [26,27]. Sporulation properties of wild *S. paradoxus* yeasts are different from those of genetically engineered laboratory strains. Investigation of different wild *S. paradoxus* strains demonstrated that even genetically identical spores were producing colonies significantly different in size [28]. This is a clear difference between wild and laboratory strains, since spores of the latter form colonies of identical size. The mechanism or possible evolutionary roles of this phenomenon are unknown, although it was suggested that laboratory strains have been artificially selected to have more synchronous spore germination [28]. Sporulation can only occur in aerobic-respiration-competent cells [25]. Increased sporulation rate often implied increased virulence, and various dsRNA viruses are involved in the modulation of this process [29,30,31,32]. In essence, dsRNA viruses of *S. cerevisiae* or *S. paradoxus* have no considerable effect on the host phenotype or growth traits, except for the killer phenotype [7,11,15]. However, in certain genetic backgrounds with impeded virus regulation system, dsRNA maintenance can lead to severe or even lethal consequences for *Saccharomyces* meiotic progeny [20].

In this work, we tested *Saccharomyces paradoxus* AML-15-66 killer strain isolated from serviceberries as a promising candidate for application in the food industry. It also serves as a model of killer yeasts prevalent in wildlife that have preserved their natural plasticity to adapt to environmental changes. Here, we examined newly derived cells that have lost M dsRNA alone, or both M and L-A dsRNAs and acquired different phenotypes. Besides converting to non-killers, cells became respiration-deficient or exhibited altered-sporulation pattern, in contrast to the wild type. This is the first whole transcriptome analysis of different phenotype *Saccharomyces* yeast cells that have been cured of viral dsRNA(s), as well as the first study analyzing transcriptional alterations of *S. paradoxus* cells with different sets of dsRNA viruses. We further provide a broader perspective of wild yeasts plasticity by comparing gene transcription profiles observed in altered-phenotype *S. paradoxus* cells that have lost viral dsRNA(s).

## 2. Materials and Methods

### 2.1. Yeast Strains and Culture Media

*Saccharomyces paradoxus* wild type AML-15-66 [L+M+] killer strain [15] bearing dsRNA SpV-L-A-66 and SpV-M-66 viruses and isogenic non-killer strains cured of M-66 or/and L-A-66 viruses with altered-sporulation pattern (Spo [L+M−], Spo [L−M−]) or exhibiting *petite* phenotype (Pet [L+M−], Pet [L−M−]) were used for transcriptome profiling. *Saccharomyces cerevisiae* α’1 (MATα *leu2-2* [*kil*-0]) was used as a sensitive strain for testing of killing phenotype [33].

YPD medium (1% yeast extract, 2% peptone, 2% dextrose) was used to cultivate yeasts and to evaluate sporulation on this medium. MBA medium (0.5% yeast extract, 0.5% peptone, 2% dextrose, 0.002% methylene blue dye, 2% agar) adjusted to pH 4.8 was used for killing phenotype assay. Minimal medium (0.17% yeast nitrogen base with ammonium sulfate and without amino acids, 2% (w/v) glucose, 2% agar) was used to evaluate prototrophy of newly generated cells. YPG medium (1% yeast extract, 2% peptone, 2% glycerol, 2% agar) was used to test the ability to use non-fermentable carbon source (glycerol) for cell growth.

### 2.2. Elimination of Viral dsRNA from Cells and Quality Control

Both L-A-66 and M-66 dsRNAs were eliminated by means of moderate heat treatment at 37 °C for 4–5 days as described in [12]. The absence of the killing phenotype was confirmed by the killing assay. The absence of the viral genome was verified by dsRNA agarose gel electrophoresis and by RT-PCR [12].

### 2.3. Killing Phenotype Assay

*Saccharomyces paradoxus* AML-15-66 killer strain and yeast colonies after the dsRNA curing were spotted onto MBA medium, seeded with sensitive *S. cerevisiae* strain α’1 (1 × 10^6^ cells/plate). Plates were incubated for 2 days at 25 °C. Non-growth zones around the colonies indicated the killing phenotype.

### 2.4. Detection of L-A-66 and M-66 dsRNAs by 2-Step RT-PCR

Total RNA extraction and subsequent dsRNA purification were performed as described in [12,34]. The absence of dsRNA(s) was confirmed by 2-step RT-PCR with specific primers for M-66 (5′-ATGTCTAAGCTGTATAATACCTCC and 5′-ATCCAGATCATGGTTGGGTT) and L-A-66 (5′-CAGGGGTTTAGGAGTGGTAGGTCTTAC and 5′-CATCTATTTCGTATGGTATTTC) that were used for both cDNA synthesis and PCR reactions. Total RNA was used as a template for cDNA synthesis carried out with RevertAid First Strand cDNA Synthesis Kit (Thermo Fisher, Vilnius, Lithuania), according to the manufacturer’s instructions. PCR reactions were conducted with DreamTaq DNA Polymerase (Thermo Fisher). PCR cycling parameters for L-A-66 detection consisted of an initial denaturation at 95 °C for 3 min; following as 30 cycles of 95 °C for 30 s, 42 °C for 30 s and 72 °C for 2 min; and a final extension step at 72 °C for 5 min. For M-66 dsRNA detection following parameters were used: 95 °C initial denaturation for 3 min, followed by 30 cycles of 95 °C for 30 s, 47°C for 30 s, 72 °C for 1 min; and a final extension step at 72 °C for 5 min. 

### 2.5. Growth Tests

The ability to grow on different media was investigated by drop tests. Wild type and cured cells were grown overnight in liquid YPD medium at 25 °C with shaking at 250 rpm. Yeasts were collected by centrifugation at 5000× *g* for 5 min and twice washed with sterile water. Cells were diluted to OD_600_ = 0.5 and four samples of 10-fold serial dilutions were prepared. Cells from dilution samples (4 µL of each) were plated on solid YPD, YPG, and minimal medium. Plates were incubated at 25 °C for 2 days.

### 2.6. Sporulation Evaluation 

Sporulation was evaluated for *S. paradoxus* cells grown in liquid and on solid YPD medium. Cells were grown on plates with YPD medium at 25 °C for 2 days. Subsequently, cells were transferred on YPD plates and, additionally, into liquid YPD medium with shaking at 250 rpm. Yeasts were incubated at 25 °C for two days. Cells grown in liquid YPD medium were collected by centrifugation at 5000× *g* for 5 min and washed with sterile water. Cells taken from YPD plates were suspended into sterile water. To evaluate sporulation frequency, 1000 cells of each type were examined. The percentage of sporulating cells was calculated as a fraction of tetrad and dyad asci in the total population of the tested cells. Sporulation frequency values are the averages of three independent experiments. Morphology of the cells was examined by light microscopy with 100-x magnification using Leica DM750 microscope combined with Leica ICC50 HD camera (Leica Microsystems, Heerbrugg, Switzerland).

### 2.7. Total RNA Extraction and Next-Generation Sequencing (NGS)

Total RNA extraction for NGS sequencing was performed as described in [12]. Total RNA was analyzed on Agilent 2100 Bioanalyzer (Agilent Technologies, Santa Clara, CA, USA). Resultant cDNA libraries were sequenced on Illumina HiSeq 2000 platform (Macrogen Inc., Seoul, Korea) in three independent biological replicates (100 nt paired-end reads configuration). Raw data are available in Gene Expression Omnibus (GEO) under accession number GSE153308.

### 2.8. NGS Data Analysis

Obtained reads were processed using CLC Genomics Workbench v. 12.0 (CLC Inc., Aarhus, Denmark). Raw reads were trimmed for sequencing adapters, ambiguous nucleotides (ambiguities 1), low quality sequences (limit = 0.01), and sequences less than 50 nt in length. Filtered reads were mapped onto the *S. paradoxus* CBS432 reference genome [35] (5531 genes in total) using the following alignment scores: mismatch cost 2; minimum length fraction 0.9; minimum identity within the mapped sequence 0.99; maximum number of best-scoring hits for a read 30. The expression values of each transcript were computed as Reads Per Kilobase of transcript per Million mapped reads (RPKM). Differential expression was determined using an exact test for two-group comparisons: wt1 [L+M+] vs. Spo [L+M−]; wt1 [L+M+] vs. Spo [L−M−]; wt2 [L+M+] vs. Pet [L+M−]; wt2 [L+M+] vs. Pet [L−M−]. Data of differentially expressed genes in *S. cerevisiae* M437 cells were obtained from [12]. Further investigation was conducted with differentially expressed genes corresponding to more than a 1.5-fold change and FDR-corrected *p*-value ≤ 0.05 [36]. 

Information provided in *Saccharomyces* Genome Database (SGD, [37]) was used for gene annotations. Gene Ontology (GO) terms were mapped by GOTermFinder [38] with default parameters. To calculate fold enrichment (F.E) we divided sample frequency (genes annotated to specific GO term) to background frequency of genes (annotated to GO term in the entire set). 

Protein-protein interaction networks were generated by employing information from STRING database (v 11.0) [39] using stringApp v. 1.5.1 [40] and represented by Cytoscape v. 3.8.0 [41]. Associations between proteins are represented by lines based on the highest confidence level (0.9).

## 3. Results

### 3.1. Phenotype Alterations of Virus-Cured Cells

*S. paradoxus* AML-15-66 K66 killer strain was isolated from spontaneous fermentation of serviceberries (*Amelanchier ovalis* Medik.) and possesses the L-A-66 and M-66 dsRNA viruses [15]. Moderate heat treatment was applied to generate cells lacking either only M-66 virus and designated as [L+M−], or both L-A-66 and M-66 dsRNA viruses, and termed [L−M−]. The absence of corresponding dsRNA(s) was confirmed by killing assay, gel electrophoresis of dsRNA, and RT-PCR (Figure S1). Consistent with the loss of M-66 virus, coding for the killer toxin, the killer phenotype was eliminated in all *S. paradoxus* [L+M−] and [L−M−] cells (Figure S1).

**Figure 1:**
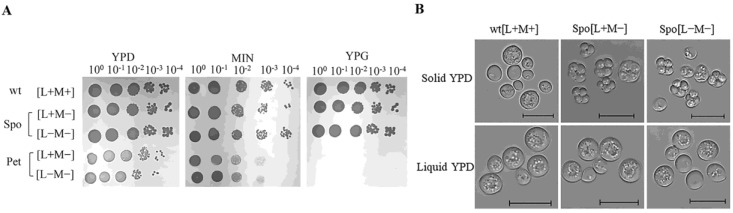
Altered phenotypes of cured *S. paradoxus* AML-15-66 cells. (**A**) Growth of *S. paradoxus* AML-15-66 cells on YPD, YPG, and minimal medium (MIN). Wild type cells carrying L-A-66 and M-66 dsRNAs, Spo (altered sporulation), and Pet (respiration-deficient) cells carrying either only L-A-66 dsRNA [L+M−], or dsRNA-free [L−M−]. Identical results were obtained with three independently isolated clones; (**B**) Light microscopy of the wild type and Spo cells grown on solid and in liquid YPD medium. Wild type cells do not form spores on solid or in liquid YPD medium. Spo [L+M−] and Spo [L−M−] sporulate on solid but not in liquid YPD medium. The scale bar is 10 µm.

The phenotype of cured cells was evaluated by several growth tests. All cells were able to grow on minimal medium deprived of amino acids (Figure 1A). This confirmed that the curing of dsRNA virus(es) had not caused auxotrophic mutations impeding the biosynthesis of essential amino acids [42]. However, cured non-killer cells demonstrated different traits when grown on YPD or YPG media (Figure 1).

The generated strains were named after the phenotype and dsRNA content. Pet cells, namely Pet [L+M−] and Pet [L−M−], exhibit *petite* phenotype, determined by small size colonies on YPD plates, incapability to grow on medium supplemented solely with glycerol as a carbon source (Figure 1A). Spo cells, namely Spo [L+M−] and Spo [L−M−], grew on YPG medium and generated colonies of similar size as the wild type (*wt*) cells (Figure 1A). Spo cells sporulated on a solid growth medium contrary to the *wt* [L+M+] strain (Figure 1B). Sporulation frequency on a solid YPD medium was 27.7 ± 1.2% for Spo [L+M−] cells and 42.9 ± 1.6% for Spo [L−M−] cells, with no sporulation observed in a liquid YPD medium. We have not detected sporulating *wt*, Pet [L+M−] and [L−M−] cells neither on solid or liquid YPD medium. Unfortunately, we were unable to detect *S. paradoxus* AML-15-66 [L+M−] and [L−M−] cells with no additional phenotype changes, besides the abolishment of the killer trait.

### 3.2. Overview of Transcriptional Changes in Pet and Spo Cells

**Figure 2:**
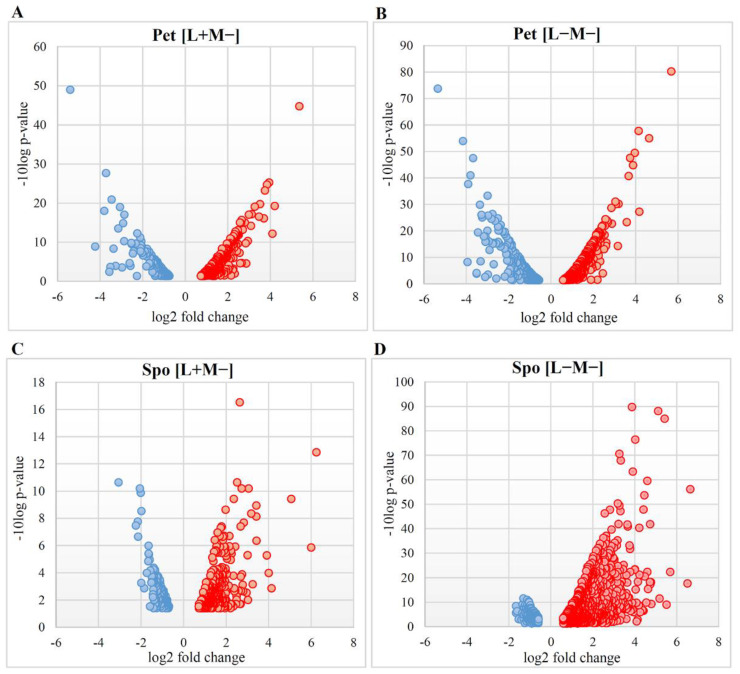
Volcano plots depicting gene expression alterations in *S. paradoxus* AML-15-66 [L+M−] and [L−M−] cells. Scattered points represent altered transcription genes: red-up-regulated, blue-down-regulated. The x-axis is the fold change for the differentially expressed genes with respect to the wild type cells, whereas the y-axis is the statistic or Log Odds, representing the probability that a gene has statistical significance in its differential expression. Differentially expressed genes in (**A**) Pet [L+M−], (**B**) Pet [L−M−], (**C**) Spo [L+M−], and (**D**) Spo [L−M−] are depicted.

To investigate gene transcription changes in newly generated strains, whole transcriptome profiling was performed. Transcription profiles of Pet [L+M−], Pet [L−M−], Spo [L+M−], and Spo [L−M−] cells were separately compared to that of the parental [L+M+] strain. Collectively, mRNA levels of 973 and 1346 individual genes were differentially regulated in cured Pet and Spo cells, respectively. Volcano plots (Figure 2) indicate the relationship between the confidence scores and the magnitude of the difference in gene expression change of samples in each set.

Altered phenotype and viral dsRNA content determined differential gene expression profiles in Pet and Spo cells (Figure 2). Transcription of more than two-thirds of up-regulated differentially expressed genes (DEGs) was altered up to four-fold, except for the Spo [L+M−] cells, where the majority of genes was positively regulated up to five-fold. Fold changes of almost all negatively regulated genes did not exceed the three-fold change limit. Thus, transcription of the majority of DEGs changed moderately. Numbers and fold change values of up- and down-regulated DEGs in Pet cells were similar, whereas those of positively regulated genes were higher than those of negatively regulated DEGs in Spo cells (Figure 2).

**Figure 3:**
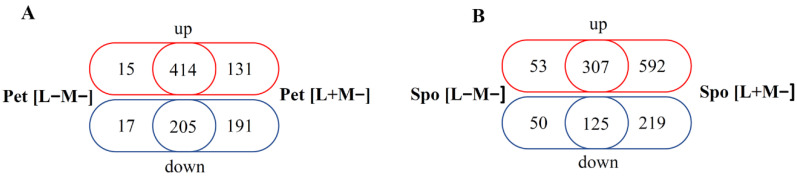
Venn diagrams representing numbers of differentially expressed genes in *S. paradoxus* AML-15-66 [L+M−] and [L−M−] cells. Numbers of up-regulated genes are in red, down-regulated-blue. Numbers of up- and down-regulated genes in (**A**) Pet [L−M−] (left) and Pet [L+M−] (right); (**B**) Spo [L−M−] (left), and Spo [L+M−] (right) cells are represented.

Elimination of viral dsRNA(s) and concomitant phenotype changes resulted in extensive gene transcription alterations in cured cells (Tables S1 and S2). In Pet [L+M−] and [L−M−] cells we have documented 941 and 651 DEGs, respectively. The majority of DEGs in Pet cells are shared since the total number of mutual DEGs (619 DEGs) is higher than the number of DEGs only found in Pet [L+M−] (322 DEGs) and Pet [L−M−] (32 DEGs) cells (Figure 3A). There were even more DEGs in Spo cells, namely 1234 and 535 DEGs in Spo [L+M−] and [L−M−] cells, respectively (Table S2 and Figure 3B). 

In contrast to Pet cells, the majority of DEGs in Spo cells were only found in Spo [L+M−] cells (811 DEGs), while 432 DEGs were common to Spo [L+M−] and dsRNA-free cells, and 103 genes were differentially expressed only in Spo [L−M−] cells (Figure 3B). There were more enhanced than suppressed genes in all derived cells (Figure 3). Even though the total number of DEGs was lower in Pet than in Spo cells, there were more shared genes between Pet [L+M−] and [L−M−] cells than between the corresponding Spo cells (Figure 3). The mRNA levels of most of the up-regulated genes were higher in Spo [L+M−] than in Spo [L−M−] cells, while in the corresponding Pet cells they were similar (Tables S1 and S2). Thus, transcriptional responses in Spo and Pet cells depend on the phenotype and the content of viral dsRNA.

Several proteins encoded by the most altered transcription genes in Pet and Spo cells are located in the cell envelope. For Pet [L+M−] and [L−M−] cells, the most enhanced genes are responsible for encoding transporters: plasma membrane ATP-binding cassette transporter Pdr5 and hexose transporter Hxt11/Hxt9, high-affinity copper transporter of plasma membrane Ctr1. Genes coding for isomaltase/alpha-glucosidase Ima2/Ima3/Ima4 and mitochondrial proteins Mto1, and Mam33 were also highly up-regulated in Pet cells (Table S1). The most suppressed genes in Pet cells were those encoding cell wall mannoproteins Tip1 and Tir1, mating pheromone alpha-factor, integral membrane protein Fig1, adhesion subunit of a-agglutinin of a-cells Aga2, and others (Table S1). Thus, the expression of many genes coding for proteins localized in the cell membrane was highly altered in Pet cells. The most increased levels of transcripts in Spo [L+M−] and [L−M−] cells were related to yeast mating. Mating pheromone encoding genes *MF(ALPHA)1* and *MF(ALPHA)2*, subunit of a-agglutinin encoding gene *AGA2*, pheromone-regulated protein encoding gene *PRM5*, genes *FUS1*, and *FUS3* encoding membrane proteins localized to the shmoo tip were among the most up-regulated genes in Spo cells (Table S2). Down-regulated genes did not show high diversity in terms of fold change values in Spo cells; abundance changes of most mRNAs did not exceed the limit of 3-fold change (Table S2). *RCK1* encoding protein kinase involved in oxidative stress response was the most down-regulated gene in Spo cells. Analysis of DEGs showing the highest changes of mRNA levels in Pet and Spo cells indicates different expression patterns and even the opposite regulation of genes involved in yeast mating signaling.

### 3.3. Functional Enrichment Evaluation of DEGs

**Figure 4:**
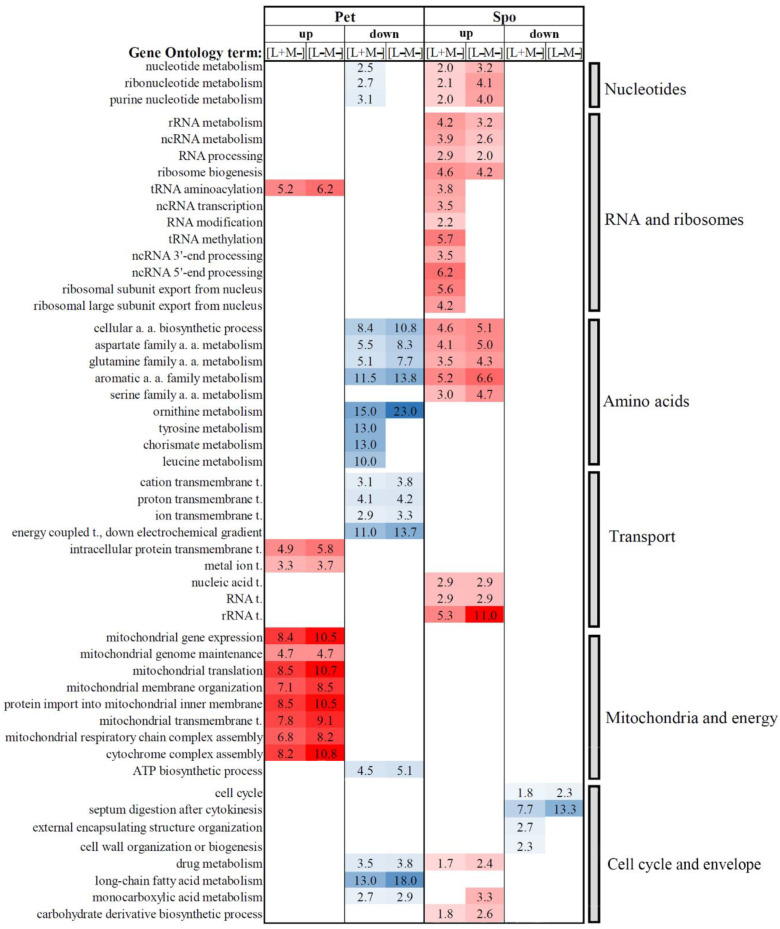
Selected statistically significant enriched gene ontology terms associated with biological processes of altered transcription genes in *S. paradoxus* AML-15-66 [L+M−] and [L−M−] cells. Fold enrichment (F.E.) values are represented by numbers. F.E. was calculated by dividing the frequency of specific gene clusters to the total frequency for each GO term, according to the data provided in Tables S3 and S4. a.a. -amino acids; t. -transport.

Gene ontology (GO) analysis was used to group DEGs. All statistically enriched “biological process” GO terms with the calculated fold enrichment (F.E.) values are presented in Tables S3 and S4. The selected GO terms were classified into the following groups, related to the cellular biological processes involving nucleotides, RNA and ribosomes, amino acids, transport, mitochondria and energy, cell cycle and cell envelope (Figure 4).

Many biological processes were altered differently in Spo and Pet cells (Figure 4, Tables S3 and S4). Metabolic processes related to nucleotides were mostly enriched by down-regulated genes in Pet [L+M−] and up-regulated genes in Spo [L+M−] and [L−M−] cells. A large group of enhanced expression genes in Spo cells represents biological processes related to RNA and ribosomes (Figure 4). In the RNA metabolism section, only genes related to tRNA aminoacylation were up-regulated in Pet [L+M−], Pet [L−M−], and Spo [L+M−] cells. In Pet cells, these genes were mostly related to tRNA aminoacylation for mitochondrial protein translation. Certain RNA-related GO terms (ncRNA transcription and processing, and RNA modification) were associated only with enhanced transcription genes in Spo [L+M−] cells (Figure 4). The abundance of transcripts linked to drug metabolic process was increased in Spo cells but decreased in Pet cells (Figure 4). Results of GO analysis illustrate a limited similarity between expression patterns of Pet and Spo cells.

Genes important to the metabolism of amino-acids-related processes were mostly represented by oppositely regulated DEGs in Pet and Spo cells (Figure 4). DEGs involved in cellular amino acid biosynthesis were down-regulated in Pet cells but up-regulated in Spo cells. Transcripts of ornithine metabolic process genes were suppressed in Pet [L+M−] and [L−M−] cells, whereas metabolic processes of tyrosine, chorismate, and leucine were enriched by down-regulated genes in Pet [L+M−] cells only. Serine family amino acid metabolic process was enriched in positively regulated genes in Spo [L+M−] and [L−M−] cells. In general, DEGs linked to the metabolism of amino acids were up-regulated in Spo cells, but down-regulated in Pet cells.

Analysis of the GO terms in the transport group suggests that transcription of genes related to transmembrane transport was mostly altered in Pet cells (Figure 4). Genes involved in transmembrane transport of positively charged ions, including energy coupled proton transport, were mainly down-regulated, while those of transmembrane transport of intracellular proteins and metal ions were up-regulated in Pet cells (Figure 4 and Table S3). In Spo cells, most genes that are related to the process of transport belong to nucleic acid transport, mainly RNA, and are enhanced (Table S4 and Figure 4). Transport linked to energy generation was suppressed, while transport of proteins and metal ions was up-regulated in Pet cells. 

Altered transcription of genes involved in various mitochondrial processes is evident in Pet cells (Figure 4 and Table S3). Positive regulation of processes related to the maintenance of mitochondria—genome maintenance, gene expression, protein translation, membrane organization, protein import, transmembrane transport, and respiratory complex assembly—is a hallmark of transcriptional responses in Pet cells (Figure 4). Suppressed genes in Pet cells are mostly related to ATP biosynthesis. Alterations of mitochondrial functioning are manifested by both, phenotypic and transcriptomic changes. 

Numerous enriched GO terms related to the cell cycle and cell envelope represent a fraction of suppressed genes in Spo cells (Figure 4 and Table S4). The abundance of transcripts of genes involved in the cell cycle and septum digestion after cytokinesis was decreased in both Spo lines. A significant number of genes related to biosynthesis and organization of external encapsulating structure and the cell wall were down-regulated in Spo cells lacking only M-66 dsRNA (Figure 4). These observations clarify differences between expression patterns in solely L-A virus maintaining and dsRNA-free Spo cells. 

Transcription profiles of viral dsRNA(s)-cured Pet and Spo cells displayed considerable disparity. Genes linked to maintenance of mitochondria were mainly up-regulated in Pet [L+M−] and [L−M−], cells, while DEGs involved in the metabolism of nucleotides, organic and amino acids, and ATP biosynthesis were down-regulated. In both types of cured Spo cells, genes associated with the metabolism of RNA, ribosomes, amino acids, and carbohydrates were positively regulated, whereas only DEGs linked to cell cycle were negatively regulated. These findings illustrate the combined impact on transcriptional responses to the elimination of viral dsRNA(s) and the change of host phenotype.

### 3.4. Interconnections of Proteins Encoded by DEGs

**Figure 5:**
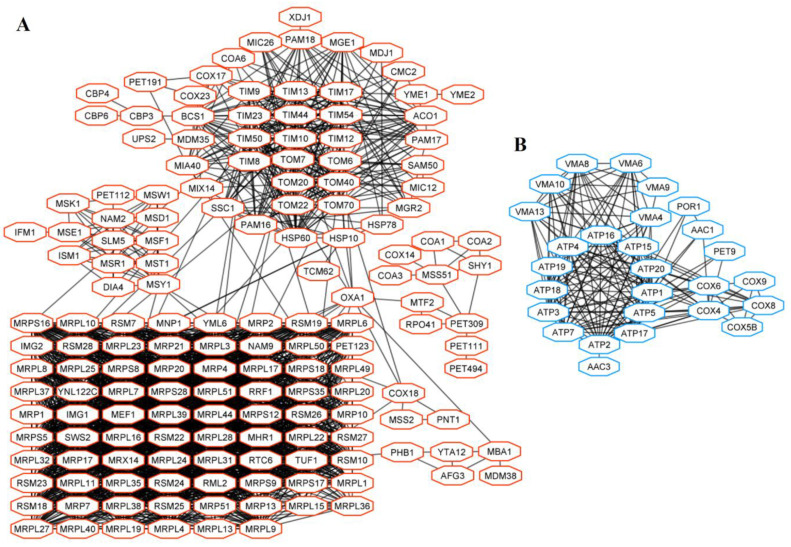
Interactions of gene products of up-regulated (**A**) and down-regulated (**B**) DEGs in Pet [L+M−] and Pet [L−M−] cells related to mitochondria and energy.

**Figure 6:**
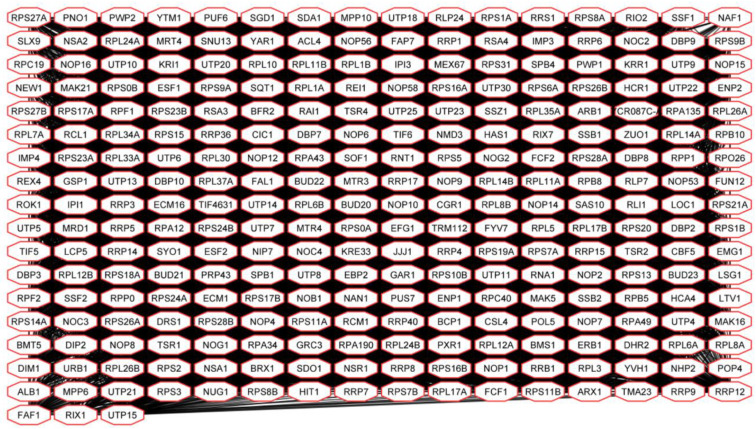
Interconnection of gene products of up-regulated genes in Spo [L+M−] and Spo [L−M−] cells involved in ribosome biogenesis.

**Figure 7:**
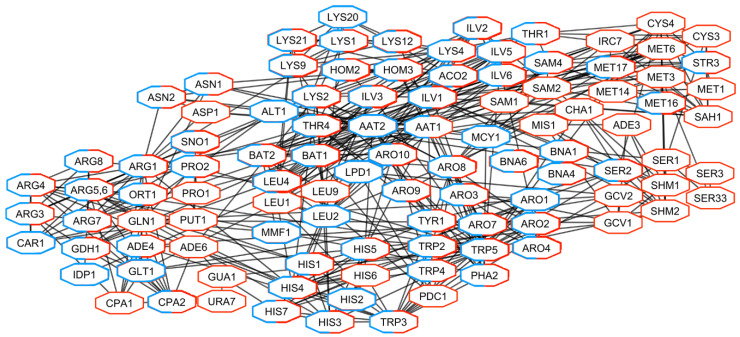
Network of gene products related to the biosynthesis of amino acids encoded by DEGs in Pet and Spo cells. Products of up-regulated genes in Spo cells-red; down-regulated in Pet cells-blue.

Changes of phenotype and viral dsRNA content affected the transcription of numerous *S. paradoxus* genes. To simplify the complex results of this work, networks of interconnected proteins encoded by selected groups of DEGs in Pet and Spo cells were generated and analyzed. Information provided in the STRING database was used to determine interconnections between gene products related to mitochondria and energy (Figure 5), ribosome biogenesis (Figure 6), and amino acids (Figure 7). 

The group of genes related to mitochondria was overrepresented in a dataset of DEGs in Pet cells (Table S3). Proteins involved in mitochondrial gene expression (e.g., Rpo41, encoding mtRNA polymerase; Mrx14, Mtf2), mitochondrial tRNA synthetases (Ism1, Nam2, Msd1), structural components of mitochondrial ribosomes (Mrp-, Mrpl-, and Mrps- proteins, forming large and small subunits of mitochondrial ribosomes) were encoded by up-regulated genes in Pet cells (Figure 5A). 

Proteins important for mitochondrial membrane organization (Mgr2, Mic12, Sam50, and others) and mitochondrial transmembrane transport (Tim- and Tom- proteins, Oxa1, Ssc1, and Pam16) were also encoded by positively regulated genes in Pet cells (Figure 5A). Down-regulated genes of Pet [L+M−] and [L−M−] cells are mostly related to ATP biosynthetic process (ATP synthase and cytochrome c oxidase genes), vacuolar membrane ATPase genes (*VMA6, -8, -9*), and encoding transporters (Aac1, Aac3, and others) (Figure 5B). The products of up- and down-regulated genes in Pet cells are interconnected at a high confidence level.

Gene products of up-regulated genes in Spo cells are involved in various RNA-related processes (Figure 4). The most numerous sub-network was formed by 275 highly interconnected proteins participating in ribosome biogenesis (Figure 6).

90S-pre ribosome components, structural elements of UTP (Utp- proteins), and MPP10 (Mpp10, Imp3, -4) complexes are encoded by genes up-regulated in Spo cells. Proteins required for ribosome maturation (Kre33, Nob1, Rix-, Nop-, and Nog- proteins), small and large ribosomal subunit export from the nucleus (protein kinase Rio2), as well as ribosomal proteins (Rpp0, Rlp24, Rpl-, and Rps- proteins) are also encoded by positively regulated DEGs in Spo cells. 

Proteins, involved in the metabolism of amino acids and mostly encoded by oppositely regulated DEGs in Pet and Spo cells, generated a highly interconnected sub-network (Figure 7). These gene products are related to aspartate family (Hom2, -3, Ilv1, Lys- proteins, and Thr1, -4), glutamine family (Arg- proteins, Cpa2, His7, Ort1, and Sno1) and aromatic amino acids (Aro-, Bna-, and Trp- proteins) metabolism (Figure 7). Products of down-regulated genes in Pet cells were involved in ornithine (Arg3, -4, -8), tyrosine (Tyr1 and Aro8), chorismate (Aro1, -3, -7), and leucine (Bat1, -2, Leu- proteins) metabolism. Proteins related to serine family amino acid metabolism (Ser-, Shm-, and other proteins) were encoded by up-regulated genes in Spo cells. Thus, removal of viral dsRNA(s) and concomitant alteration of phenotype changed the transcription of genes encoding highly interconnected proteins that are at least involved in the maintenance of mitochondrial and energetic functions, biosynthesis of ribosomes, and cellular amino acids.

## 4. Discussion

In this study, concomitant phenotype changes and dsRNA(s) elimination occurring in native *S. paradoxus* yeasts induced by moderate heat treatment were documented and investigated. Temperature is an important abiotic factor contributing to environmental changes occurring in the yeast-driven industry, thus, moderate heat treatment was chosen for curing cultures of dsRNA viruses. A wild type phenotype conversion into *petite* or altered sporulation pattern may be explained by the fact that *S. paradoxus* is less thermo-tolerant than *S. cerevisiae* [43,44]. Pet cells form reduced-size colonies on YPD medium and cannot utilize non-fermentable carbon source glycerol for growth, indicating that these cells have defects in the oxidative phosphorylation pathway, resulting in growth suppression upon the exhaustion of dextrose [45,46]. The rise of the *petite* phenotype is likely unrelated to the dsRNA loss, as it was demonstrated for *S. cerevisiae* [16,18]. Respiration deficiency in Pet cells could have occurred due to the loss of total mitochondrial DNA or defects in mitochondrial and/or chromosomal genes [47,48]. Pet cells were unable to undergo sporulation since it demands aerobic respiration [25]. The nature of the sporulation pattern of Spo cells remains elusive. Wild *S. paradoxus* yeasts have asynchronous sporulation patterns [28], whereas in *S. cerevisiae*, variations in sequences of several genes are associated with sporulation efficiency alterations [49,50,51]. Changes in sporulation pattern may be provoked by alterations in signaling and/or metabolism since sporulation is induced by the lack of nitrogen and fermentable carbon sources, and the presence of non-fermentable carbon source resulting in the arrest in G1 phase [52]. However, enhanced sporulation rate is associated with viral-dsRNA-mediated virulence in fungi [29,30,31,32]. In the case of dsRNA viruses of yeasts, the amount of L-A-encoded Gag protein decreases due to the suppressive action of the M virus [20]. Certain combinations of disrupted *NUC1* and *SKI3* genes, and maintenance of L-A and M dsRNAs can result in severe or even lethal phenotypes in sporulating cells [20]. Maintenance of the L-A virus alone in sporulating diploids completely lacking *NUC1* was associated with the generation of respiration-deficient progeny, demonstrating the possible negative effect of virus propagation [20]. Thus, in certain circumstances, the elimination of viral dsRNA(s) may salvage the host from the manifestation of detrimental phenotypes. 

In this work, we documented complex transcriptional patterns in either respiration-deficient (Pet) or altered sporulation status (Spo) *S. paradoxus* cells with different dsRNA content. Surprisingly, elimination of only M-66 dsRNA in Pet and Spo cells resulted in a higher number of DEGs than elimination of both L-A-66 and M-66 dsRNAs. It might be related to the parasitic relationship of the M satellite and the L-A virus, since the M dsRNA maintenance requires proteins encoded by the L-A genome [53], or is a result of combined effects of phenotypic change and viral dsRNA. Elimination of the M dsRNA may facilitate the L-A replication and even increase the L-A dsRNA copy number [54] and it can contribute to the transcriptional changes in the [L+M−] cells. There were more positively than negatively regulated genes in Pet and Spo cells. Pet [L+M−] and [L−M−] cells showed a higher number of shared DEGs than Spo [L+M−] and [L−M−] (Figure 3). Thus, transcriptomic response in the Pet background is more similar between [L+M−] and [L−M−] cells than in the Spo background, presumably because of the different metabolic capabilities of these cell types. It suggests that dsRNA content has a more prominent role in gene transcription of respiration-competent than respiration-deficient cells.

DEGs in Pet and Spo cells were related to numerous biological processes, including metabolism and biogenesis of nucleotides, RNA, ribosomes, amino acids, carbohydrates, and lipids (Figure 4, Tables S3 and S4). Genes of altered transcription were also linked to cellular transport, mitochondria, cell cycle, and cell envelope (Figure 4, Tables S3 and S4). GO analysis confirmed that biological processes related to mitochondria prevail in Pet cells, while those linked to RNA and ribosomes dominate in Spo cells. Transcription of genes related to mitochondria and ATP biosynthesis appeared dysregulated in Pet cells, probably because of the inactivation of aerobic respiration in these cells, and acts to compensate for this loss. Metabolic processes of amino acids were oppositely regulated in Pet and Spo cells, probably because of the differences in energy generation. 

Evident transcriptional alterations of yeast mating-related genes were observed in Pet and Spo cells. Expression patterns of alpha factor and a-agglutinin indicate that little to no expression of these genes occurs in Pet cells, slightly more—in the wild type cells, and the most—in Spo cells. The differences between sporulation efficiency were prominent in plate-grown cells (Figure 1B). Although sporulation in a liquid growth medium was not observed in the tested cells (Figure 1B), transcription profiles were linked to the cells of different mating types, located within the spores [52]. Membrane and cell wall reorganization also occur during spore formation, when a stress-resistant wall is formed around the spore [52]; thus, expression of genes associated with these processes, including stress response, may be also associated with sporulation. It implies that regulation of mating genes is different in the wild type, Pet and Spo cells, and may be linked to both the phenotype and dsRNA content.

Transcriptional alterations of genes linked to mitochondrial functions in Pet cells are related to the impaired oxidative phosphorylation. Enhanced expression of hexose transporters in Pet cells may aid the import of fermentable carbon sources into the cell to compensate for the respiration defects. Upregulation of *PDR* family genes (*PDR3, PDR5, PDR15, PDR16*, and *PDR10*) in Pet cells can be linked to the *petite* phenotype, in agreement with the previously published data [55,56]. Transcriptomic pattern of Pet cells is highly similar to that of the cells lacking the mitochondrial genome [56], suggesting that phenotype plays a major role in shaping the transcriptomic profile of Pet cells. 

Down-regulated genes in Spo cells (Figure 4 and Table S4) represented processes involved in the cell cycle. A recent study also reported negative regulation of genes related to mitotic cell cycle upon exposure of yeast cells cured of dsRNAs to nitazoxanide, when compared to the parental killer strain [57]. These findings suggest that dsRNA viruses may play a significant role in the host transcriptional regulation in different growth conditions. During sporulation, the expression of more than a thousand genes is altered [58,59] and expression patterns depend on the yeast strain [59]. Out of 477 genes, differentially expressed during sporulation and specified in the recent publication [59], only 35 were differentially expressed in Spo [L+M−] and/or [L−M−] cells. Down-regulation of *IME2* in both types of Spo cells and up-regulation of *RME1* in Spo [L+M−] cells suggest that sporulation is repressed in Spo cells since *IME2* codes for protein kinase acting in sporulation process [60] and *RME1* is encoding the repressor of meiosis [61]. Thus, besides sporulation, transcriptional alterations in Spo cells seem to be linked to other factors such as dsRNA content.

Expression of genes related to the killer phenotype maintenance and killer toxin susceptibility was altered in Pet and Spo cells. *MAK5* gene was up-regulated in Pet [L+M−] and Spo [L+M−] cells (Tables S1 and S2). *MAK16* and *MAK21* were also up-regulated in Spo [L+M−] cells (Table S2). All these MAK genes are related to biogenesis of 60S ribosomal subunit and are critical for L-A dsRNA maintenance [62]. Another gene related to ribosome biogenesis and positively regulated in Spo [L+M−] cells is *KRE33*, which is also involved in the killer toxin resistance [63]. Gene encoding Ski7, a component of the mRNA degrading Ski complex [64], is down-regulated in Spo [L+M−] cells. Deletion of *SKI* genes is known to promote super killer phenotype [65], thus, down-regulation of these genes may act in a similar manner to increase viral dsRNA abundance in a host cell. *SEC14*, *SEC53*, and *SEC63*, encoding proteins involved in processing and secretion of the killer toxin [13], were up-regulated in Spo [L+M−] cells. Results of McBride et al. [13] study have shown that transcription of *SEC* genes was only slightly altered upon dsRNA infection. Dataset of up-regulated genes in Spo [L+M−] cells was the largest in terms of the total number of DEGs and showed the highest number of altered transcription genes related to killer maintenance. Out of 73 genes associated with susceptibility to K66 toxin [15], expression of only 19 genes was altered in Pet and Spo cells, whereas out of 52 genes involved in the resistance to K66 toxin, expression of 22 genes was changed in these cells.

To investigate whether there are any similarities between transcriptional responses related to dsRNA elimination in different yeast strains, we have compared DEGs in *S. paradoxus* AML-15-66 Spo, Pet (this work), and *S. cerevisiae* M437 cells bearing either L-A dsRNA alone or no virus [12]. A part of altered transcription genes and GO terms of DEGs were shared in these cells. Genes related to amino acid biosynthesis were down-regulated in M437 [L−M−] and [L+M−], similarly to AML-15-66 Pet [L−M−] and [L+M−] cells. DEGs linked to ribosome biogenesis and assembly were positively regulated in M437 [L+M−] and AML-15-66 Spo cells. Genes related to ATP biosynthesis and mitochondrial electron transport were down-regulated in M437 [L+M−] and in AML-15-66 Pet cells. 

**Figure 8:**
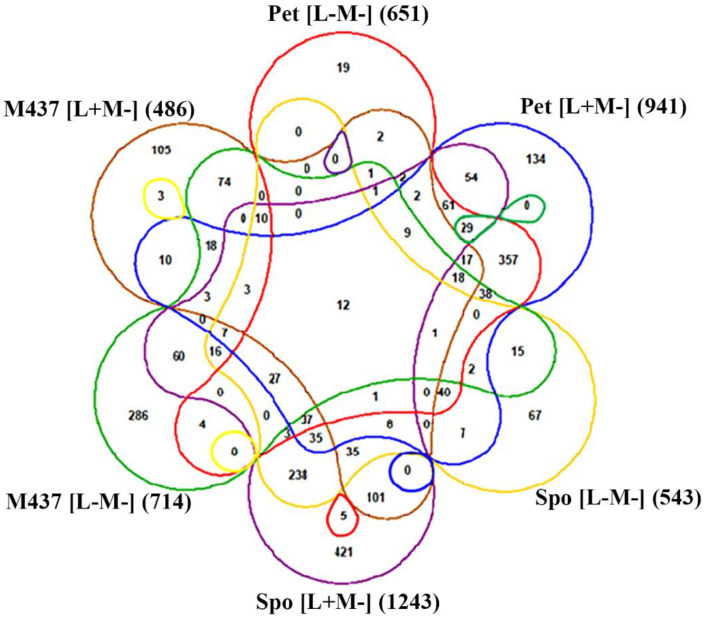
Six-way Venn diagram representing the distribution of DEGs between *S. paradoxus* AML-15-66 Spo [L+M−], Spo [L−M−], Pet [L+M−], Pet [L−M−] and *S. cerevisiae* M437 [L+M−] and M437 [L−M−] cells. Up- and down-regulated genes were not analyzed separately. Numbers represent the quantity of shared DEGs between the datasets. Data from [12] were used for the analysis of DEGs in M437 cells.

DEGs in *S. paradoxus* AML-15-66 Spo [L+M−], Spo [L−M−], Pet [L+M−], Pet [L−M−] (compared to AML-15-66 [L+M+]) and *S. cerevisiae* M437 [L+M−] and M437 [L−M−] (compared to M437 [L+M+]) were analyzed (Figure 8). There were only twelve shared DEGs between all these cell types. At least six of them are related to the metabolism of amino acids (*ARO10, ARG1, -5,6, TRP2, -5*, and *THR1*), while *SNO1* and *SNZ1* encode proteins forming glutaminase complex, and *BNA1* gene product is required for *de novo* NAD biosynthesis. Genes encoding cell wall mannoprotein Tir1, membrane protein Izh1, and glucose transporter Hxt3 were also among shared DEGs. Since viral dsRNAs encode Gag, Gag-Pol, and killer toxin proteins, its elimination induces changes in the expression of genes involved in the metabolism of amino acids and other processes that appear to be host- and virus-specific.

## 5. Conclusions

In this study, gene transcription alterations in two different phenotypes *S. paradoxus* cells with alternative sets of dsRNA viruses were evaluated for the first time. Cured cells showed respiration-deficient and altered sporulation phenotypes, and diverse transcriptional responses combined with the effects of dsRNA loss. Overall, modest magnitude gene expression changes were documented. Products of differentially expressed genes were highly interconnected and linked to various cellular processes including the metabolism of RNA, nucleotides, amino acids, carbohydrates, and lipids. Our findings reveal distinct action of dsRNA viruses in the regulation of gene transcription in hosts of different phenotypes. They also highlight the effect of transcriptional and phenotypic variations of the wild killer yeasts, arising upon environmental stress that can be encountered during industry-related processing.

## Supplementary Materials

The following are available online at https://www.mdpi.com/2076-2607/8/12/1902/s1, Figure S1: Quality control of dsRNA content in *S. paradoxus* AML-15-66 cells, Table S1: Altered transcription genes in Pet cells, Table S2: Altered transcription genes in Spo cells, Table S3: GO terms of altered transcription genes in Pet cells, Table S4: GO terms of altered transcription genes in Spo cells.
