# Supplementary material for: Saccharomyces paradoxus Transcriptional Alterations in Cells of Distinct Phenotype and Viral dsRNA Content
Source: Microorganisms. 2020 Nov 30;8(12):1902. doi: 10.3390/microorganisms8121902 (PMC7761358; doi:10.3390/microorganisms8121902)
Supplement: Supplementary file 1 [file microorganisms-08-01902-s001.zip › FigS1.pdf]

Supplementary material: *Saccharomyces paradoxus* transcriptional alterations in cells of distinct phenotype and viral dsRNA content

Bazilė Ravoitytė, Juliana Lukša, Vyacheslav Yurchenko, Saulius Serva and Elena Servienė

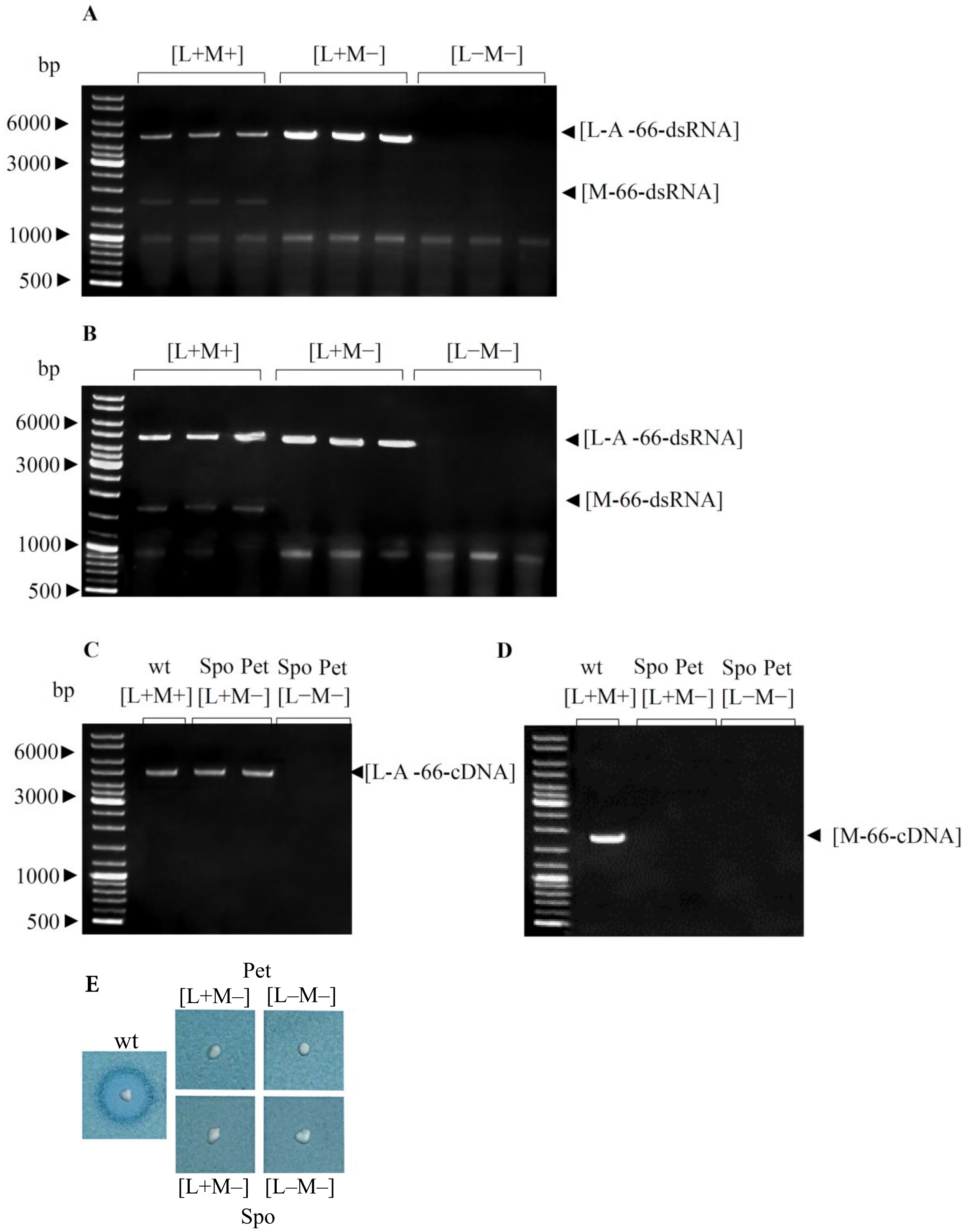

Figure S1. Quality control of dsRNA content in *S. paradoxus* AML-15-66 cells. Tree independent replicates of each type were tested. (**A**) dsRNA content in the wild type [L+M+], Pet [L+M–], and Pet [L–M–] cells. (**B**) dsRNA content in the wild type [L+M+], Spo [L+M–], and Spo [L–M–] cells. (**C**) L-A-66-cDNA and (**D**) M-66-cDNA correspond to the cDNA products of L-A-66 and M-66 viruses. Molecular weight (bp) marker GeneRuler DNA Ladder Mix (Thermo Fisher, Vilnius, Lithuania). (**E**) Killing phenotype of virus bearing and virus-free cells against sensitive *S. cerevisiae* α'1 cells.
